# Identification of a Mosaic *BMPR1A* Pathogenic Variant in Juvenile Polyposis Syndrome: A Case Study and Its Impact on Cancer Screening

**DOI:** 10.1155/crig/6578711

**Published:** 2025-11-09

**Authors:** Kara Rogen, Lisa Boardman, Megan Bird

**Affiliations:** ^1^Department of Clinical Genomics, Mayo Clinic, Rochester, Minnesota, USA; ^2^Division of Gastroenterology and Hepatology, Mayo Clinic, Rochester, Minnesota, USA

**Keywords:** *BMPR1A*, juvenile polyposis syndrome, mosaicism

## Abstract

Juvenile polyposis syndrome (JPS) (MIM: 174900) is a rare genetic disorder characterized by multiple benign, hamartomatous polyps, and an increased risk for colorectal and gastric cancer. It is caused by pathogenic variants in *SMAD4* and *BMPR1A*. We present the findings of a mosaic *BMPR1A* pathogenic variant in a 57-year-old patient with newly diagnosed colon cancer and a history of polyps, which were later discovered to be JPS polyps. The variant was first identified in a blood sample at approximately 15% allele frequency. Subsequent genetic testing performed on gDNA from cultured fibroblasts found this variant to be present at very low levels (< 10%). The finding of this *BMPR1A* variant in two sample types, as well as the history of JPS polyps, supports a diagnosis of JPS due to a mosaic *BMPR1A* pathogenic variant. This diagnosis affects cancer screening recommendations for our patient and his relatives. Our case highlights the need for recognition and workup of potentially mosaic cases and for universal germline genetic testing for patients with colorectal cancer.

## 1. Introduction

Juvenile polyposis syndrome (JPS) (MIM: 174900) is a rare genetic condition with autosomal dominant inheritance. It is characterized by multiple benign hamartomatous polyps called juvenile polyps, which have a distinctive histologic and pathologic appearance. These polyps are found primarily in the colorectum but can also be found in the stomach and small bowel. Individuals with JPS have an increased risk for developing colorectal and gastric malignancy. Current National Comprehensive Cancer Network (NCCN) [1] guidelines suggest up to a 50% lifetime risk for colorectal cancer and up to a 21% risk for gastric cancer, if multiple gastric polyps are present. A clinical diagnosis of JPS can be made in an individual with five or more juvenile polyps in the colorectum, two or more juvenile polyps throughout the gastrointestinal tract, or any number of juvenile polyps in an individual with a family history of JPS.

JPS is caused by pathogenic variants in the *SMAD4* gene (located on chromosome 18q21) and in the *BMPR1A* gene (located on chromosome 10q22-23), which play a role in the bone morphogenetic protein/transforming growth factor beta pathway and thus are involved in the maintenance of colonic mucosa. Pathogenic variants in *SMAD4* and *BMPR1A* are identified in 45%–60% of individuals with a clinical diagnosis of JPS [2–6]. Up to 25% of cases are due to a de novo variant [3]. Individuals with pathogenic variants in *SMAD4* have higher risks of gastric polyps and gastric cancer than individuals with pathogenic variants in *BMRP1A* [2, 3, 5, 6]. Additionally, most individuals with pathogenic variants in *SMAD4* have hereditary hemorrhagic telangiectasia (HHT) (MIM: 175050).

Constitutional mosaicism occurs when a postzygotic event changes the genetic composition of some cells of the embryo. It is likely underrecognized. With improvements to genetic testing technology, such as next-generation sequencing, constitutional mosaicism may be identified more frequently [7, 8]. Clinical presentation for individuals with mosaicism is variable and may be milder than the heterozygous state. However, the variant could be present in the germline and therefore transmitted to offspring, who would be expected to be heterozygous.

Constitutional mosaicism has been reported as a disease mechanism for colorectal cancer and polyposis susceptibility genes. It is believed that up to 20% of individuals with de novo familial adenomatous polyposis (MIM: 175100) are mosaic for an *APC* pathogenic variant. Constitutional mosaicism has also been reported in other colorectal cancer and polyposis susceptibility genes, such as *MLH1*, *MSH2*, *PTEN*, and *STK11* [4, 7]. There are reports of patients with JPS due to a mosaic *SMAD4* pathogenic variant [4, 9–11]. There are also two reported cases of patients with mosaic deletions of chromosome 10q23, encompassing *BMPR1A*, *PTEN*, and other genes. These patients presented with a combined phenotype of JPS and *PTEN* hamartoma tumor syndrome (MIM: 158350) or “Infantile” JPS (MIM: 612242) [12, 13]. We also identified a report of three individuals with pathogenic variants in *BMPR1A*, found at 10%–30% allelic frequency on a blood or saliva sample. Testing of additional tissues was not performed, and the variants were assumed to be associated with clonal hematopoiesis [8]. Otherwise, to our knowledge, there have been no reports of individuals with a mosaic pathogenic variant in the *BMPR1A* gene.

We present the case of a patient with constitutional mosaicism of a *BMPR1A* pathogenic variant. This case highlights the importance of recognizing mosaic presentation of JPS, as well as the need for universal genetic testing of all patients with colorectal cancer.

## 2. Clinical Report

Our patient is a 57-year-old who was assigned male at birth. He underwent a surveillance colonoscopy, showing invasive, moderately differentiated adenocarcinoma arising in a background of tubular adenoma with low- and high-grade dysplasia. Mismatch repair screening by immunohistochemical staining showed intact MLH1, MSH2, MSH6, and PMS2 in the tumor. The patient also had tubular adenomas with low-grade dysplasia in the sigmoid and descending colon. An esophagogastroduodenoscopy (EGD) was performed concurrently and showed a normal stomach and duodenum. Prior colonoscopy at age 53 showed multiple polyps, and the patient was recommended to have repeat colonoscopy in 3 years. Pathology and procedure reports from the prior colonoscopy were not available at the time of our initial consultation.

The patient's family history is not highly suggestive of a hereditary cancer syndrome. He is the youngest of six children, with the eldest brother having a diagnosis of leukemia. Both parents passed away in their late 70s. The patient's maternal grandmother was diagnosed with breast cancer or another cancer in her 80s. The patient's maternal grandfather was diagnosed with colon cancer in his early 90s. The patient notes limited information regarding his paternal relatives' health history. In light of recent changes to the NCCN guidelines, our clinic offers genetic testing to all patients diagnosed with colorectal cancer, regardless of age at diagnosis, family history, and mismatch repair tumor screening results.

The patient elected to pursue a 77-gene hereditary cancer panel with concurrent RNA analysis through Ambry Genetics Laboratory. Whole blood was collected in an EDTA and a PAXgene tube. Genomic deoxyribonucleic acid (gDNA) and ribonucleic acid (RNA) were isolated and quantified from the patient's sample using standardized methods. RNA was converted to complementary DNA (cDNA) by reverse transcriptase polymerase chain reaction (RT-PCR). Bait capture methods were used for sequence enrichment of the targeted coding exons and adjacent intronic nucleotides. Custom-designed biotinylated probes (IDT X-Gen Lockdown) covering the coding regions of tested genes were hybridized overnight to capture libraries and captured with streptavidin beads (Life Technologies). Captured libraries were subsequently amplified and prepared for sequencing on NovaSeq using the SP flow cell (Illumina). Initial data processing and base calling are performed using the NextSeq Control Software (NCS) and Real-Time Analysis (RTA) NCS.

Sequences are aligned to hg19 reference genomes, and variants are called using the third-party software Genome Analysis Toolkit (GATK) followed by annotation via an internally developed pipeline. In general, the lab requires minimal coverage of 250x reads across the regions analyzed. Variants in regions complicated by pseudogene interference, variant calls not satisfying depth of coverage and variant allele frequency quality thresholds, and potentially homozygous variants are verified by Sanger sequencing. Variants with a *Q* score ≤ 30 and an allele fraction < 10% are typically filtered out. However, additional pipeline and manual review may be completed on a case-by-case basis related to findings and phenotype [14].

**Figure 1:**
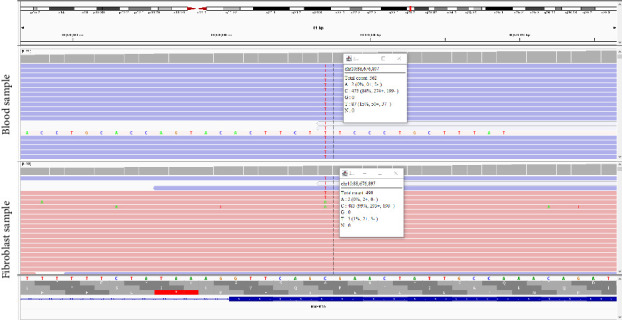
Top: Integrative Genomics Viewer (IGV) output, showing the total number of reads at nucleotide position 682 and the number of reads showing the pathogenic variant in the patient's blood sample. Bottom: IGV output from the patient's gDNA specimen from cultured fibroblasts.

The patient's results showed a variant in the *BMPR1A* gene, NM_004329.2: c.682C > T (p.R228^∗^), at approximately 15% allele frequency (seen in 87 of 562 reads; see Figure 1). The patient was counseled regarding the possibility of constitutional mosaicism for JPS. Further testing was recommended via testing of his offspring and testing on gDNA from cultured fibroblasts for clarification. The patient elected to proceed with a skin punch biopsy. Local anesthesia was given via 1% lidocaine with epinephrine. A 4-mm punch biopsy was obtained from the right flank by a dermatologist, and the specimen was placed in fibroblast biopsy transport media (T115). The specimen was cut into smaller pieces, treated with collagenase, and placed in a tissue culture flask with Chang and MEM alpha-medium, 20% fetal bovine serum, and antibiotics. The cultures were trypsinized into one to three T25 tissue culture flasks or one to two T75 tissue culture flasks. gDNA was extracted and sent to Ambry Genetics for analysis of the *BMPR1A* gene.

While awaiting the patient's genetic test results from cultured fibroblasts, our team requested pathology slides from the patient's previous colonoscopy. Our internal pathology review found three inflammatory polyps consistent with either an inflammatory polyp or a hamartomatous polyp of juvenile inflammatory type in the transverse colon and rectum. A tubular adenoma with low-grade dysplasia was also reported. Juvenile polyps can have dysplasia, making them difficult to distinguish from adenomas. In addition, adenomas and low-grade dysplasia have been reported in individuals with JPS [3, 5, 6]. This further supported the diagnosis of JPS due to a mosaic *BMPR1A* variant; however, even with these findings, the patient did not quite meet clinical criteria for a diagnosis of JPS.

Genetic testing on gDNA from cultured fibroblasts was reported as negative by the lab. However, a special comment on the test report notes that the NM_004329.2:c.682C > T (p.R228^∗^) variant was detected at levels below the reporting threshold and could not be orthogonally confirmed. The variant was detected at approximately 1% variant allele fraction in gDNA from cultured fibroblasts (seen in 5 of 490 reads; see Figure 1).

## 3. Discussion

The NM_004329.2:c.682C > T (p.R228^∗^) variant is located in Exon 7 of the *BMPR1A* gene, creating a premature stop codon. The resulting protein is expected to result in loss of function through premature protein truncation or nonsense-mediated decay. This variant has been previously reported in several individuals with a clinical diagnosis of JPS. Following ACMG/AMP guidelines, the variant was classified as pathogenic [15] and submitted to ClinVar as such (https://www.ncbi.nlm.nih.gov/clinvar/variation/142735/).

Upon receiving the initial test report, showing this variant at 15% variant allele frequency, we considered several potential explanations for the lower allele frequency. Constitutional mosaicism was the top of the differential. Another possible explanation was that the test detected a somatically acquired *BMPR1A* variant from circulating tumor cells. The patient's colon cancer was a clinical stage IIIA, so it seemed unlikely the origin of this variant was circulating colon cancer cells. The patient also had a recent, normal complete blood count; it therefore seemed unlikely that the origin of this variant was circulating cells from an undiagnosed hematologic malignancy. We also considered the possibility of clonal hematopoiesis of indeterminate potential (CHIP) or aberrant clonal expansion (ACE). Pathogenic variants in *BMPR1A* have only been reported in CHIP in one publication [8]. In this study, the variants were assumed to be related to CHIP due to a variant allele fraction lower than 50%. Confirmation using a second sample was not performed, and constitutional mosaicism cannot be excluded. A diagnosis of CHIP therefore seemed unlikely for our patient since *BMPR1A* variants have not been reported in CHIP.

With the finding of the *BMPR1A* variant at 15% variant allele frequency in blood and low levels (∼1% variant allele frequency) in gDNA from cultured fibroblasts, as well as the finding of three JPS polyps, the patient was counseled as having JPS due to a mosaic *BMPR1A* pathogenic variant. The patient was informed his lifetime risk of developing cancer may be lower than someone who is heterozygous for a *BMPR1A* pathogenic variant; however, the percent mosaicism in other tissues (i.e., colorectum and stomach) is unknown. With the *BMPR1A* variant only being seen at 1% allele frequency in gDNA from cultured fibroblasts, it is possible that the specimen included gDNA from white blood cell contamination. Even with this possibility, we feel that a diagnosis of mosaic JPS is appropriate for our patient due to the other clinical findings, as well as having a low suspicion for CHIP or circulating tumor cells as potential explanations for the initial mosaic result found on blood.

With the diagnosis of mosaic JPS, the patient is recommended to undergo regular GI screening. Following his colon cancer diagnosis, the patient will undergo a colonoscopy in 1 years' time. Depending on findings, a repeat colonoscopy is recommended for 1–3 years, eventually increasing to a 5-year interval follow-up. We informed the patient that colonoscopy should occur every 1–3 years, per current recommendations from the NCCN for patients with JPS. This interval is determined by the number of polyps found, their size, and the pathology. The patient was also recommended to undergo upper endoscopy every 1–3 years, with the interval being determined by polyp size, number, and pathology.

Genetic testing is recommended for the patient's blood relatives, particularly his offspring. The risk for his offspring to have the same *BMPR1A* variant may be as high as 50%. Since the percent mosaicism in the patient's germ cells may be less than 50%, it is unclear what the true risk to his offspring is. However, if they did inherit the variant from their father, they would be heterozygous for the *BMPR1A* variant, which may have a more severe presentation. Prior to genetic testing, the patient was counseled that his children should start colonoscopy at age 40. However, individuals with a pathogenic variant in *BMPR1A* are recommended by the NCCN to have a baseline colonoscopy and upper endoscopy at age 12–15. If polyps are found, these procedures should be repeated in 2-3 years. If normal, the screening outlined in the above paragraph resumes at age 18. In some cases of significant polyposis, high-grade dysplasia, GI bleeding and/or anemia, and/or other symptoms, colectomy or gastrectomy may be considered [2, 3, 5, 6]. If the patient's offspring test positive for the *BMPR1A* pathogenic variant, screening would start at a much younger age than what had been recommended prior to the receipt of these genetic test results. If his offspring test negative for the familial variant, they can follow general population colon cancer screening guidelines, starting at age 45.

Since the patient's parents are deceased, he was counseled that his siblings could consider testing for this variant out of an abundance of caution. The *BMPR1A* variant likely occurred as a postzygotic event, meaning his siblings would not be at risk of having the variant. There is a small possibility that the variant was inherited from one of the patient's parents and that a postzygotic somatic reversion event occurred. However, the patient's family history is not suggestive of an inherited *BMPR1A* pathogenic variant.

This case highlights the importance of identifying constitutional mosaicism for hereditary cancer predisposition syndromes. The identification of the *BMPR1A* pathogenic variant in our patient leads to increased cancer screening recommendations for both our patient and potentially his relatives. Although the *BMPR1A* variant was identified on the patient's initial genetic test using a blood sample, the diagnosis of JPS was not felt to be confirmed until the variant was identified in a different tissue source, highlighting the need for further workup when mosaic variants are identified. To our knowledge, this is the first case reported of a patient with constitutional mosaicism for a *BMPR1A* pathogenic variant. Similar findings may become more frequent with the use of NGS for genetic testing.

We recognize that differences in next-generation sequencing platforms at different genetic testing institutions may have varying abilities to detect low-level mosaicism. In addition, policies for the reporting of low-level variants may vary among genetic testing laboratories. Therefore, while recognition of mosaic genetic conditions is imperative, a mosaic variant may not be routinely reported at all genetic testing institutions due to the above limitations.

This case also supports the recommendation for universal germline genetic testing of all patients with colorectal cancer. Current NCCN guidelines state to “consider germline multigene panel testing for Lynch syndrome and other hereditary cancer syndromes for all individuals with colorectal cancer aged ≥ 50 years at diagnosis,” even if tumor mismatch repair screening is normal and there is not a strong family history of cancer. Testing is more strongly recommended for individuals diagnosed with colorectal cancer under the age of 50, with a strong family history of cancer, abnormal mismatch repair tumor screening, and/or a personal history suggestive of a specific syndrome. Had we followed typical guidelines for genetic testing, testing may not have been offered to this patient, and the *BMPR1A* pathogenic variant may not have been identified until additional polyps and/or cancer were later identified in the patient and/or his offspring.

In conclusion, the finding of constitutional mosaicism may become a more frequent finding on genetic testing. Additional workup should be performed to confirm the diagnosis of constitutional mosaicism through testing DNA from additional tissue sources and testing blood relatives. These patients may present with less severe disease than patients who have heterozygous variants. Therefore, they may not meet classic guidelines for genetic testing, arguing the case for universal testing of patients diagnosed with colorectal cancer.

## Data Availability

Data sharing is not applicable to this article as no new data were created or analyzed in this study.
